# Supplementary material for: Multiunit In Vitro Colon Model for the Evaluation of Prebiotic Potential of a Fiber Plus D-Limonene Food Supplement
Source: Foods. 2021 Oct 7;10(10):2371. doi: 10.3390/foods10102371 (PMC8535099; doi:10.3390/foods10102371)
Supplement: Supplementary file 1 [file foods-10-02371-s001.zip › foods-1386805-supplementary.pdf]

Table S1. Baseline values of VOCs related to prebiotic activity in mg/kg.

VOCs	Baseline (mg/kg)
Acetic acid	0.288 ± 0.083
Propanoic acid	0.141 ± 0.027
Butanoic acid	0.129 ± 0.002
Pentanoic acid	0.123 ± 0.003
Hexanoic acid	0.000 ± 0.000
Heptanoic acid	0.000 ± 0.000
Octanoic acid	0.000 ± 0.000
Nonanoic acid	0.000 ± 0.000
n-Decanoic acid	0.000 ± 0.000
Pentanoic acid, 3-methyl	0.142 ± 0.058
Butanoic acid, 3-methyl	0.245 ± 0.024
Propanoic acid, 2-methyl	0.304 ± 0.011
Indole	16.250 ± 5.515
1H-Indole, 3-methyl	0.541 ± 0.112

Table S2. Primers pairs employed for PCR and qPCR reactions and quantifications.

Bacterial taxa	Target	Sequence 3'-5'	Base pairs	MT*	Reference
Eubacteria	*V3-V4 16S*	Eub518-R: ATTACCGCGGCTGCTGG	147	57.6	Lane et al, 1991
		Eub338-F: ACTCCTACGGGAGGCAG		63.5	
Firmicutes	*V3-V4 16S*	Firm934-F: GGAGYATGTGGTTTAATT	300	60.5	Guo et al, 2008
		Eub338-R: ACTCCTACGGGAGGCAG		63.5	
Bacteroidetes	*V3-V4 16S*	Bact934-F: GGARCATGTGGTTTAATT	250	58.9	Guo et al, 2008
		Bact1060-R: AGCTGACGACAACCATG		59.4	
Lactobacillales	*V3-V4 16S*	F-Lac: GCAGCAGTAGGGAATCT	340	59.8	Walter et al, 2001
		R-Lac: GCATTYCACCGCTACACA		58.3	
Bifidobacteriaceae	*RecA*	RecA-F: CGTYTCBCAGCCGGAYA	220	60.3	Masco et al, 2006
		RecA-R: CCARVGCRCCGGTCATC		59.2	
Enterobacteriaceae	*V3-V4 16S*	Enterob-F: TGCCGTAACTTCGGGAG	450	64.2	Bartosh et al, 2004
		Enterob-R: TCAAGGACCAGTGTTCAG		60.3	
Clostridium group I	*V3-V4 16S*	ClosI-F: TACCHRAGGAGGAAGCCAC	148	54.6	Bartosh et al, 2004
		ClosI-R: GTTCTTCCTAATCTCTACGCAT		53.0	
Clostridium group IV	*V3-V4 16S*	ClosIV-F: TTAACACAATAAGTWATC	400	58.1	Goldberg et al, 2013
		ClosIV-R: ACCTTCCTCCGTTTTGTC		57.9	
Escherichia coli	*FtsZ*	EcFtsZ-F: GGTATCCTGACCGTTGCT	250	59.4	Zhou & Helmstetter, 1994
		EcFtsZ-R: ATACCTCGGCCCAGAACT		57.3	
Akkermansia muciniphila	*V3-V4 16S*	AkM1: CAGCACGTGAAGGTGGG	327	63.5	Guo et al, 2016
		AkM2: CCTTGCGGTTGGCTTCA		59.4	
Faecalibacter prausnitzii	*V3-V4 16S*	Fprau223-F: GATGGCCTCGCGTCCGA	199	63.7	Wang et al, 1996
		Fprau420-R: CCGAAGACCTTCTTCCTC		58.8	

*Melting Temperature

Table S3. MANOVA categorical descriptors for the volatilome, categorized for the type of matrix. % of contribution of VOCs descriptors significant amid the food matrices.

**VOCs**	**FOS %**	**FLS %**	**P value**
Acetaldehyde	100.00	0.00	0.021196
2-Butenal, 2-methly, (E)	0.00	100.00	0.004190
Benzaldehyde	3.74	96.26	0.046352
Furancarboxaldehyde	25.70	74.30	0.034243
Benzaldehyde, 3,5-dimethyl	100.00	0.00	0.004397
Ethyl alcohol	81.06	18.94	0.008725
3-Buten-1-ol, 3-methyl	3.74	96.26	0.049540
Isotridecanol	100.00	0.00	0.041860
1-Hexanol, 2-ethyl	4.92	95.08	0.022025
1,6-Octadien-3-ol, 3,7-dimethyl	5.00	95.00	0.000774
1-Octanol	4.91	95.09	0.000070
1-Nonanol	18.48	81.52	0.000010
p-Menth-1-en-8-ol	0.00	100.00	0.030519
3-Buten-2-one, 3-methyl	100.00	0.00	0.006583
Hexanone, 5-methyl	100.00	0.00	0.047791
2,4-Pentanedione	100.00	0.00	0.036135
4-Isopropylcyclohexanone	0.00	100.00	0.000550
p-Menthanone	0.00	100.00	0.002113
Acetophenone	21.30	78.70	0.027490
Ethanone, 1-(3-methylphenyl)	0.00	100.00	0.000015
Dimethyl trisulfide	0.00	100.00	0.000003
Benzenamine, 3-(trifluoromethyl)	100.00	0.00	0.040909
Phenol	6.47	93.53	0.000177
Benzenemethanol, 4-(1-methylethyl)	0.00	100.00	0.004301
1H-Inden-5-ol, 2,3-dihydro	100.00	0.00	0.001414
1H-Indole, 5-methyl-phenyl	100.00	0.00	0.009905

Table S4. MANOVA categorical descriptors for the volatilome, categorized for the time of fermentation. % of contribution of VOCs descriptors significant amid the time points.

VOC	6 h %	18 h %	24 h %	P value
Acetaldehyde	14.55	65.56	19.89	0.043432
Butanal, 2-methyl	11.28	45.71	43.01	0.015944
Hexanal	0.00	0.00	100.00	0.010567
Benzaldehyde	50.28	47.56	2.16	0.033290
1-Propanol	11.37	41.75	46.88	0.013369
1-Butanol	12.28	44.90	42.82	0.006751
1-Hexanol	17.10	37.00	45.90	0.012795
Benzyl alcohol	6.98	7.03	85.99	0.013642
2-Butanone	6.22	22.78	71.00	0.042579
Butanedione	4.34	23.55	72.11	0.003344
2-Pentanone	6.23	25.34	68.43	0.016105
2-Hexanone	1.20	6.25	92.55	0.005513
2-Butanone, 3-hydroxy	0.87	2.80	96.33	0.010938
p-Menthanone	8.96	45.72	45.32	0.043980
Acetophenone	1.73	2.08	96.20	0.011561
Thiourea	1.77	6.15	92.08	0.011358
Benzothiazole	8.10	18.83	73.07	0.007840
Phenol, 4-methyl	20.89	29.00	50.11	0.046953
Butylated hydroxytoluene	23.15	34.70	42.14	0.043657

Figure S1. Quantification heatmap of total VOCs.

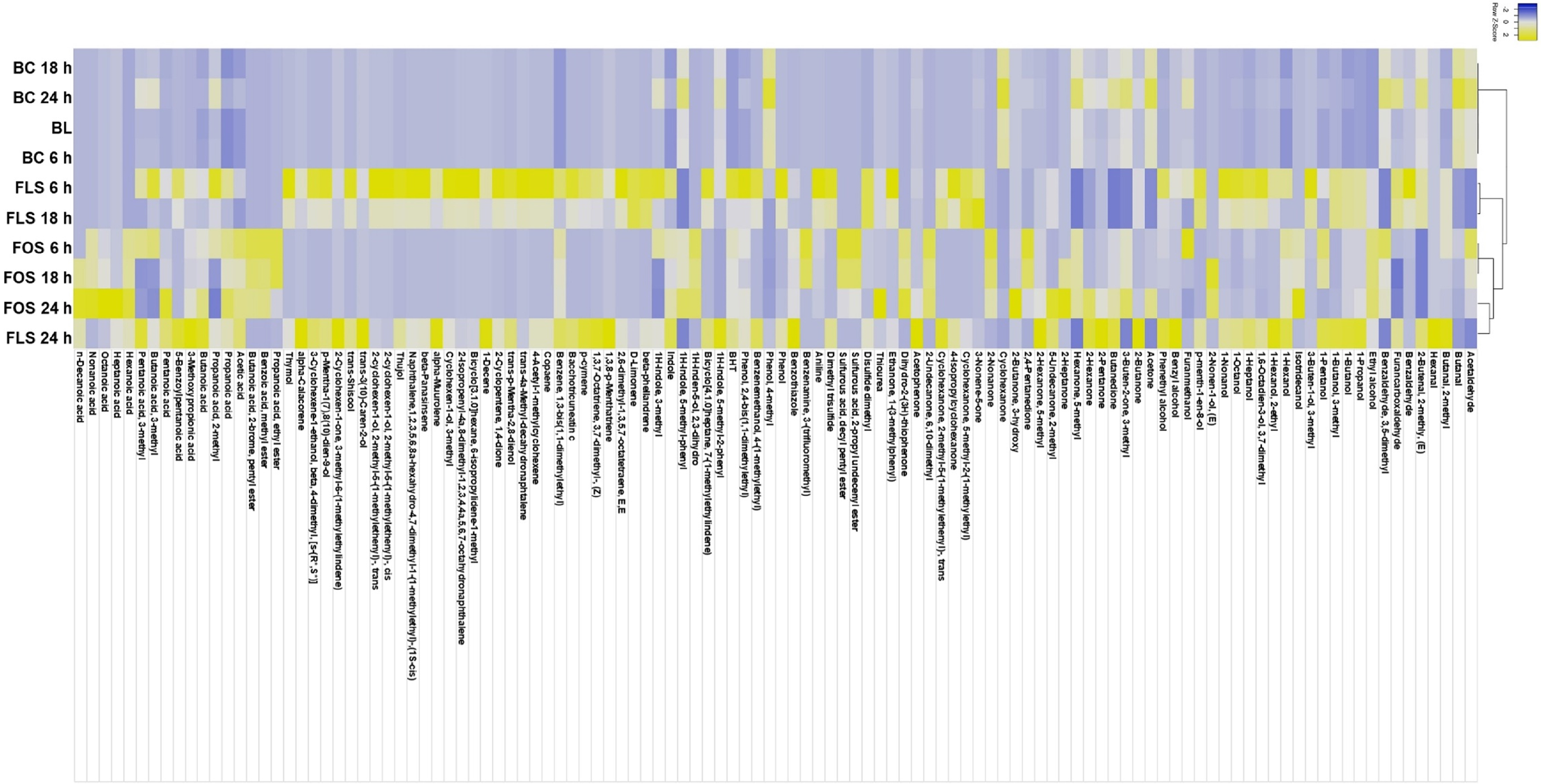

Figure S2. Significance of Spearman rank correlations.

Spearman Rank Order Correlations (Spreadsheet4)
MD pairwise deleted
Marked correlations are significant at p <,05000

Variable	Firmicutes	Bacteroidetes	Lactobacillales	Bifidobacteriaceae	Enterobacteriaceae	Clostridium group I	Clostridium group IV	A muciniphila	E. coli	F. prausnitziii
Acetic acid	-0,235396	0,828571	0,942857	0,771429	-0,753702	-0,314286	0,371429	0,542857	-0,88571	0,885714
Propanoic acid	-0,235396	0,771429	0,885714	0,657143	-0,753702	-0,085714	0,085714	0,371429	-0,94286	0,771429
Butanoic acid	-0,147122	0,885714	0,942857	0,771429	-0,811679	-0,142857	-0,085714	0,542857	-1,00000	0,828571
Pentanoic acid	0,088273	0,714286	0,771429	0,771429	-0,985611	-0,257143	-0,142857	0,257143	-0,82857	0,828571
Hexanoic acid	-0,117698	0,771429	0,885714	0,828571	-0,840668	-0,485714	0,485714	0,485714	-0,77143	0,942857
Heptanoic acid	-0,441367	0,657143	0,828571	0,600000	-0,550782	-0,428571	0,600000	0,485714	-0,71429	0,771429
Octanoic acid	-0,441367	0,657143	0,828571	0,600000	-0,550782	-0,428571	0,600000	0,485714	-0,71429	0,771429
Nonanoic acid	-0,562775	0,394665	0,637536	0,394665	-0,462031	-0,516100	0,698253	0,212512	-0,51610	0,637536
n-Decanoic acid	-0,179124	0,811679	0,927634	0,811679	-0,808824	-0,405840	0,434828	0,521794	-0,84067	0,927634
Pentanoic acid, 3-methyl	0,411943	0,085714	-0,142857	0,142857	0,202920	-0,142857	-0,200000	0,485714	0,25714	-0,085714
Butanoic acid, 3-methyl	0,235396	-0,828571	-0,942857	-0,771429	0,753702	0,314286	-0,371429	-0,542857	0,88571	-0,885714
Propanoic acid, 2-methyl	0,323669	-0,600000	-0,771429	-0,657143	0,637748	0,600000	-0,714286	-0,428571	0,60000	-0,828571
Indole	-0,029424	0,771429	0,828571	0,714286	-0,898645	-0,085714	-0,257143	0,314286	-0,94286	0,771429
1H-Indole, 3-methyl	0,441367	-0,657143	-0,828571	-0,600000	0,550782	0,428571	-0,600000	-0,485714	0,71429	-0,771429

Supplementary Reference

1. Lane, D.J.; Harrison, Jr. A.P.; Stahl, D.; Pace, B.; Giovannoni, S.J.; Olsen, G.J.; Pace, N.R. Evolutionary relationships among sulfur- and iron-oxidizing eubacteria. *J Bacteriol*, **1992**, *174(1)*, 269-278. https://doi:10.1128/jb.174.1.269-278.1992
2. Guo, X.; Xia, X.; Tang, R.; Zhou, J.; Zhao, H.; Wang, K. Development of a real-time PCR method for *Firmicutes* and *Bacteroidetes* in faeces and its application to quantify intestinal population of obese and lean pigs. *Lett Appl Microbiol*, **2008**, *47(5)*, 367-73. https://doi.org/10.1111/j.1472-765X.2008.02408.x
3. Walter, J.; Hertel, C.; Tannock, G.W.; Lis, C.M.; Munro, K.; Hammes, W.P. Detection of *Lactobacillus*, *Pediococcus*, *Leuconostoc*, and *Weissella* species in human feces by using group-specific PCR primers and Denaturing Gradient Gel Electrophoresis. *Appl Environ Microbiol*, **2001**, *67(6)*, 2578-2585. https://doi.org/10.1128/AEM.67.6.2578-2585.2001
4. Masco, L.; Ventura, M.; Zink, R.; Huys, G.; Swings, J. Polyphasic taxonomic analysis of *Bifidobacterium animalis* and *Bifidobacterium lactis* reveals relatedness at the subspecies level: reclassification of *Bifidobacterium animalis* as *Bifidobacterium animalis* subsp. *animalis* subsp. *nov*. and *Bifidobacterium lactis* as *Bifidobacterium animalis* subsp. *lactis* subsp. *nov*. *Int J Syst Evol Microbiol*, **2004**, *54*, 1137–1143. https://doi.org/10.1099/ijs.0.03011-0
5. Bartosch, S.; Fite, A.; Macfarlane, G.T.; McMurdo, M.E. Characterization of bacterial communities in feces from healthy elderly volunteers and hospitalized elderly patients by using real-time PCR and effects of antibiotic treatment on the fecal microbiota. *Appl Environ Microbiol*, **2004**, *70(6)*, 3575-81. https://doi.org/10.1128/AEM.70.6.3575-3581.2004
6. Goldberg, E.; Amir, I.; Zafran, M.; Gophna, U.; Samra, Z.; Pitlik, S.; Bishara, J. The correlation between *Clostridium difficile* infection and human gut concentrations of *Bacteroidetes* phylum and clostridial species. *Eur J Clin Microbiol Infect Dis*, **2014**, *33(3)*, 377-83. https://doi.org/10.1007/s10096-013-1966-x
7. Zhou, P.; Helmstetter, C.E. Relationship between ftsZ gene expression and chromosome replication in *Escherichia coli*. *J Bacteriol*, **1994**, *176*, 6100–6106. https://doi.org/10.1128/jb.176.19.6100-6106.1994
8. Guo, X.; Zhang, J.; Wu, F.; Zhang, M.; Yi, M.; Peng, Y. Different subtype strains of *Akkermansia muciniphila* abundantly colonize in southern China. *J Appl Microbiol*, **2016**, *120*, 452–459. https://doi.org/10.1111/jam.13022
9. Wang, R.F.; Cao, W.W.; Cerniglia, C.E. PCR detection and quantitation of predominant anaerobic bacteria in human and animal fecal samples. *Appl. Environ. Microbiol*. 1996, *62*, 1242–1247, https://doi.org/10.1128/AEM.62.4.1242-1247.1996.
